# Left Renal Vein Division for Juxtarenal Aortic Exposure: Influence on Renal Function and Role of the Communicating Lumbar Vein

**DOI:** 10.1007/s00268-022-06480-6

**Published:** 2022-03-26

**Authors:** Andreas Selberherr, Marta Mari, Markus Klinger, Christopher Burghuber, Wolf Eilenberg, Bernd Gollackner, Christoph Neumayer, Christoph Domenig

**Affiliations:** 1 Division of Vascular Surgery, Department of General Surgery, Medical University, Währinger Gürtel 18-20, 1090 Vienna, Austria; 2 Department of General and Visceral Surgery, Evangelisches Krankenhaus Wien, Hans-Sachs-Gasse 10-12, 1180 Vienna, Austria; 3 Department of Vascular Surgery, Azienda Ospedaliera SS. Antonio e Biagio e Cesare Arrigo, 15121 Alessandria, Italy

## Abstract

**Background:**

In this study, we evaluate the outcome of renal function in patients undergoing juxtarenal abdominal aortic aneurysm repair with or without division of the left renal vein with special focus on the role of the communicating lumbar vein.

**Methods:**

A retrospective analysis of prospectively collected data of 110 patients undergoing elective juxtarenal abdominal aortic aneurysm repair between 2000 and 2018 was performed.

The demographic characteristics and comorbidities were reviewed in detail and the renal function was analysed pre- and post-operatively. The cohort of patients was split into group A (left renal vein divided) and B (left renal vein mobilised). Group A was further sub-analysed regarding the presence of a communicating lumbar vein on preoperative imaging data (group A+ = vein present, group A− = no communicating lumbar vein present).

**Results:**

The patients were matched well regarding their demographic characteristics and comorbidities. In the analysis of renal function, no statistically significant difference could be detected between group A and B. In the sub-analysis of group A, the group with a communicating lumber vein (group A+) turned out to have a significantly better renal function in the long term (sCrea 0.87 vs. 1.51; *p* = 0.016).

**Conclusion:**

Ligation of the left renal vein is a safe procedure in surgery of juxtarenal aortic aneurysms regarding the outcome of the renal function. A communicating lumbar vein between the left renal vein and the left ascending lumbar vein seems to play a key role to provide venous drainage after division of the left renal vein.

## Introduction

Juxtarenal aortic aneurysms are rare with an incidence of 2.2/100,000 and account for 10.5% of abdominal aortic aneurysms [1]. During open repair of juxtarenal aortic aneurysms, the left renal vein (LRV) is dissected and frequently divided to achieve adequate exposure of the juxtarenal aortic segment. According to the literature, the left renal vein division (LRVD) rate ranges from 1.3 to 18.8% during this procedure [2,3]. After LRVD, a significant increase in serum creatinine values is reported one day and one month after an elective juxtarenal aneurysm repair [4]. Also, West et al. [5] documented a significant association between renal impairment and LRVD after elective aortic aneurysm repair. Moreover, AbuRahma et al. [6] described increased perioperative levels of creatinine. Wang et al. found decreased glomerular filtration rates (GFR) and, as observed by Mehta et al. [2], increased creatinine values immediately after surgery which recovered in the long-term. Samson [7] described no changes in serum creatinine and GFR during the first postoperative days, at discharge and at 12 months postoperatively compared to preoperative values.

The literature seems quite contradictory and no reasonable explanation for deteriorated renal function after LRVD in one group of patients vs. normal function in the other group has been identified so far.

Typically, the venous system is characterised by a large number of collateral vessels, which provide alternative pathways to allow blood flow if vessels are obstructed or ligated like the left renal vein during juxtarenal aneurysm repair. Gonadal, adrenal and left ascending lumbar veins are the venous collaterals of the left renal vein [8–10]. A communicating vein, linking the ascending lumbar vein to the left renal vein is described by some authors [8,11]. The incidence of this communicating lumbar vein (CLV) in contrast-enhanced CT studies is approximately 35% [8]. In case of division or obstruction of the LRV, venous collaterals (including the CLV) and the left renal stump may enlarge due to the diversion of blood flow from the left kidney [8]. However there is no data on changes in renal function taking into consideration this anatomical variation [12].

The aim of this study was to investigate the perioperative renal function of patients undergoing elective open juxtarenal aortic aneurysm repair. The follow-up period was two years. The effects of LRVD were compared to mobilization only or reconstruction of the LRV. In addition, a sub-analysis on a potentially protective effect of a communicating lumbar vein on the renal function after LRVD was performed. The images shown in this manuscript are provided in agreement with the respective patient.

## Materials and methods

From January 2000 to December 2018, 110 patients underwent elective aortic open surgery for juxtarenal abdominal aortic aneurysm repair at our institution. Data were prospectively collected and retrospectively analysed. Patients with infrarenal abdominal aortic aneurysms und patients with ruptured abdominal aortic aneurysms were excluded from analysis. Patients were divided in two groups: group A (*n* = 81 patients) with suprarenal aortic cross clamping and LRVD; group B (*n* = 29 patients) with suprarenal aortic cross clamping and LRV mobilisation without division. The cross-clamping times were comparable between the groups. The left renal vein was divided close to the inferior vena cava, far from the collateral veins (adrenal, gonadal and communicating vein to the left ascending lumbar vein) to preserve venous drainage. If the LRV was mobilised, the suprarenal and gonadal veins were divided. The patients were matched well according to risk factors, interestingly there was a trend towards smoking in group A. In group B coronary artery disease was (insignificantly) more common (Tables 1, 2).

**Table 1:** Demographic characteristics of patients (group A vs. group B)

Characteristics	Total cohort (*n* = 110)	Suprarenal clamp + LRV division patients (group A; *n* = 81)	Suprarenal clamp + LRV mobilization patients (group B; *n* = 29)	*p*-Value
Sex, male, *n* (%)	90 (81.8%)	65 (80.2%)	25 (86.2%)	0.475
Age, years, mean ± SD	69.5 ± 8.7	68.8 ± 9.3	71.4 ± 6.6	0.179
ASA, median	3.04	3.04	3.03	0.985
Dialysis, *n*, (%)	1 (0.9%)	1 (1.2%)	0	0.552
CKD stage ≥ 3b, *n* (%)	14 (12.7%)	9 (11.1%)	5 (17.5%)	0.525
Dyslipidemia, *n* (%)	69 (62.7%)	48 (59.3%)	21 (72.4%)	0.212
Diabetes mellitus, *n* (%)	17 (15.5%)	14 (17.3%)	3 (10.3%)	0.380
Hypertension, *n* (%)	94 (85.5%)	68 (84.0%)	26 (89.7%)	0.459
Smoker, *n* (%)	66 (60.0%)	53 (65.4%)	13 (44.8%)	0.053
CAD, *n* (%)	46 (41.8%)	30 (37.0%)	16 (55.2%)	0.091
Peripheral artery disease, *n* (%)	36 (32.7%)	29 (35.8%)	7 (24.1%)	0.255
Pulmonary disease, *n* (%)	34 (30.9%)	23 (28.4%)	11 (37.9%)	0.345

*CKD* chronic kidney disease; *CAD* coronary artery disease

**Table 2:** Demographic characteristics of patients (group A+ vs. group A−)

Characteristics	Total cohort (*n* = 81)	Suprarenal clamp + LRV division patients + communicating lumbar vein present (group A+ ; *n* = 25)	Suprarenal clamp + LRV division patients + communicating lumbar vein missing (group A−; *n* = 56)	*p*-Value
Sex, male, *n* (%)	65 (80.2%)	17 (68.0%)	45 (80.4%)	0.225
Age, years, mean ± SD	68.8 ± 9.3	68.9 ± 7.7	68.8 ± 10.0	0.959
ASA, median	3.04	2.84	3.13	0.064
Dialysis, *n*, (%)	1 (1.2%)	0	1 (1.8%)	0.507
CDK stage ≥ 3b, *n* (%)	9 (11.1%)	2 (8.0%)	7 (12.5%)	0.114
Dyslipidemia, *n* (%)	48 (59.3%)	16 (64.0%)	32 (57.1%)	0.567
Diabetes mellitus, *n* (%)	14 (17.3%)	3 (12.0%)	11 (19.6%)	0.407
Hypertension, *n* (%)	68 (84.0%)	21 (84.0%)	47 (84.0%)	0.994
Smoker, *n* (%)	53 (65.4%)	15 (60.0%)	38 (67.9%)	0.498
CAD, *n* (%)	30 (37.0%)	9 (36.0%)	21 (37.5%)	0.899
Peripheral artery disease, *n* (%)	29 (35.8%)	8 (32.0%)	21 (37.5%)	0.638
Pulmonary disease, *n* (%)	23 (28.4%)	5 (20.0%)	18 (32.1%)	0.269

*CKD* chronic kidney disease; *CAD* coronary artery disease

Furthermore, to identify a possible variable to stratify the risk for deterioration of renal function after suprarenal cross clamping and division of the LRV, we focused on the anatomy of the left renal vein in the preoperative contrast enhanced CT scan images. Special attention was given to the presence of a communicating lumbar vein between the left renal vein and the left ascending lumbar vein that could serve as an additional drainage pathway after division of the left renal vein.

Group A (*n* = 81 patients) was further divided into two subgroups: group A+ (communicating lumbar vein present on CT-scan preoperatively) with *n* = 25 patients and group A− (no communicating lumbar vein present on CT-scan preoperatively) with *n* = 56 patients.

For both comparisons of groups A and B as well as of groups A+ and A−, patient characteristics, risk factors and perioperative parameters including intensive care unit stay and 30 day mortality were analysed. The following parameters were calculated: mean values of serum creatinine preoperatively and at postoperative days 10 and 15 as well as at discharge and 12 and 24 months after the operation. In addition to that, the GFR was calculated using the CDK-EPI formula preoperatively, at discharge and 12 as well as 24 months postoperatively [3,13,14]. Dichotomous variables were compared using the chi-square test or Fisher’s exact test where indicated, continuous variables were compared using the student’s *t*-test or Mann Whitney *U* Test as appropriate. For all statistical analysis a *p*-value <0.05 was considered significant. All statistical analyses were performed using the statistical analysis software package SPSS (26.0, IBM, Boston, USA version).

### Ethical statement

All procedures performed in this study were in accordance with the ethical standards of the institutional research committee and with the 1964 Helsinki Declaration and its later amendments. The present study was approved by the local ethics review board with the reference number EC 1309/2017. The study is registered on ClinicalTrials.gov, trial identification number: NCT05054972.

## Results

Detailed patients’ comorbidities and demographics are described in Tables 1 and 2. No significant differences were found between groups A and B (Table 1) or groups A+ and A− (Table 2).

The evaluation of the renal outcome showed similar results between groups A and B during the early postoperative course (days 1–15) as well as in the long-term follow-up (12 and 24 months postoperatively), details are described in Table 3.

**Table 3:** Serum creatinine (sCr) and eGFR values (group A vs. group B)

Characteristics	Total cohort (*n* = 110)	Suprarenal clamp + LRV division patients (group A; *n* = 81)	Suprarenal clamp + LRV mobilization patients (group B; *n* = 29)	*p*-Value
*Preoperative*				
sCr (mg/dl)	1.2	1.17	1.27	0.271
eGFR (ml/min/1.73m^2^)	73.32	72.31	61.18	0.699
*Postoperative day 10*				
sCr (mg/dl)	1.46	1.57	1.17	0.086
*Postoperative day 15*				
sCr (mg/dl)	1.48	1.59	1.18	0.119
*Discharge*				
sCr (mg/dl)	1.38	1.46	1.16	0.126
eGFR (ml/min/1.73m^2^)	67.39	66.91	68.73	0.726
*12 months postoperative*				
sCr (mg/dl)	1.2	1.16	1.24	0.646
eGFR (ml/min/1.73m^2^)	70.61	72.35	65.2	0.303
*24 months postoperative*				
sCr (mg/dl)	1.29	1.31	1.17	0.619
eGFR (ml/min/1.73m^2^)	73.92	74.01	73.50	0.975

The sub-analysis of group A showed that 25 patients (group A+) had a communicating lumbar vein detectable in the preoperative CT scan (diameter ≥ 2 mm), whereas in 56 patients this vein was absent (group A−).

With regard to renal function between group A+ and group A−, both sCr and eGFR had an initial overlap during the early postoperative period and after 12 months. Slightly lower mean sCrea values were observed in group A+ , but no statistical significance could be detected (1.01 vs. 1.23; *p* = 0.262). After 24 months of follow-up patients who did not present with a communicating lumbar vein in the preoperative CT scan showed a significant deterioration of renal function: sCr at 24 months was 0.87 mg/dl in group A+ and 1.51 mg/dl in group A−, *p* = 0.016; the eGFR at 24 months was 96.2 ml/min/1.73 m^2^ in group A+ and 64.3 mL/min/1.73m^2^ in group A−, *p* = 0.041. Detailed results are described in Table 4.

**Table 4:** Serum Creatinine (sCr) and eGFR values (group A+ vs. group A−)

Characteristics	Total cohort (*n* = 81)	Suprarenal clamp + LRV division patients + communicating lumbar vein present (group A+ ; *n* = 25)	Suprarenal clamp + LRV division patients + communicating lumbar vein missing (group A−; *n* = 56)	*p*-Value
*Preoperative*				
sCr (mg/dl)	1.17	1.12	1.19	0.471
eGFR (ml/min/1.73m^2^)	72.31	79.64	69.04	0.063
*Postoperative day 10*				
sCr (mg/dl)	1.57	1.36	1.66	0.295
*Postoperative day 15*				
sCr (mg/dl)	1.59	1.46	1.64	0.591
*Discharge*				
sCr (mg/dl)	1.46	1.37	1.49	0.605
eGFR (ml/min/1.73m^2^)	66.91	72.18	64.56	0.209
*12 months postoperative*				
sCr (mg/dl)	1.16	1.01	1.23	0.262
eGFR (ml/min/1.73m^2^)	72.35	76.13	70.71	0.487
*24 months postoperative*				
sCr (mg/dl)	1.31	0.87	1.51	0.016 sig.
eGFR (ml/min/1.73m^2^)	74.01	96.23	64.29	0.041 sig.

Two patients died peri-operatively and both belonged to group A. They were both ASA 4 patients, affected by chronic ischaemic cardiac disease, chronic obstructive pulmonary disease, second stage chronic kidney disease and peripheral artery disease. One patient had an intraoperative cardiac arrest, the other patient died after 19 days due to congestive heart failure. Despite this, 30 day mortality was insignificantly different between the groups (2.5% in group A and 0% in group B, *p* = 0.15). No significant difference was observed concerning the mean duration of the ICU stay (6.14 days in group A and 4.2 days in group B, *p* = 0.19).

The sub-analysis of group A showed a 30 day mortality similar between the groups (3.6% in group A− and 0% in group A+ , *p* = 0.16) and no statistically significant difference emerged in mean ICU stay between group A+ and A− (8.64 days in group A+ and 5.18 days in group A−, *p* = 0.22).

No patient was lost to follow-up.

## Discussion

Despite the advances and increasing frequency of endovascular techniques performed, juxtarenal and complex aortic aneurysms frequently require open surgical treatment. At our institution, we prefer the transperitoneal approach. For adequate exposure of the pararenal aortic segment LRV division or extensive mobilisation of the LRV is required.

In our series, we compared renal function outcomes between patients who required division of the LRV (group A) with patients in whom the aortic cross clamp could be placed safely without division but mobilisation of the LRV (group B). We subsequently performed a sub-analysis of group A with regard to the presence of a communicating lumbar vein (CLV) as detected on preoperative CT imaging (group A+ : CLV present; group A−: CLV not present).

We observed an elevation of serum creatinine values throughout postoperative day 15, but no significant differences between groups A and B or A+ and A−. This observation is consistent with early postoperative data from Wang et al. [15] and Samson et al. [7]. We therefore cannot confirm the findings of Huber et al. West et al. and AbuRahma et al. who described a relevant deterioration of renal function after division of the left renal vein, compared to mobilization only [2,4–6]. Interestingly the collective of patients in these studies was not as uniform as in our study. Huber et al. [4], Mehta et al. [2] and Wang et al. [15] included a large number of patients with ruptured aortic aneurysms into their analyses (29%, 39% and 37% of patients; respectively). In patients with ruptured aortic aneurysms shock due to blood loss represents an important factor contributing to the deterioration of renal function. In our opinion, these patients should be analysed as an individual cohort.

In other studies, mixed cohorts of patients who received both supra- and infrarenal aortic clamps were analysed negligent of the position of the aortic cross clamp [2,4,7,16]. Using this approach the effect of LRVD on renal function in patients with suprarenal aortic cross clamping cannot be determined sufficiently. Therefore, findings of the aforementioned studies need to be interpreted with caution [2,4,17]. A recent publication compared renal outcome with regard to LRVD versus mobilisation only in a cohort in which 68,4% of the patients included underwent infrarenal aortic cross clamping [16]. In our opinion it seems impossible to draw conclusions concerning the impact of LRVD on renal function when the position of the aortic cross clamp is not standardised.

A third factor that affects renal function, besides emergency surgery and the exact position of the aortic cross clamp is additional treatment to the renal arteries (bypass, reimplantation, endarterectomy) [5].

For these aforementioned reasons, we only included patients undergoing aortic cross clamping at the same level (suprarenal) who were not operated under emergency conditions and who did not receive additional treatment of the renal arteries.

**Fig. 1:**
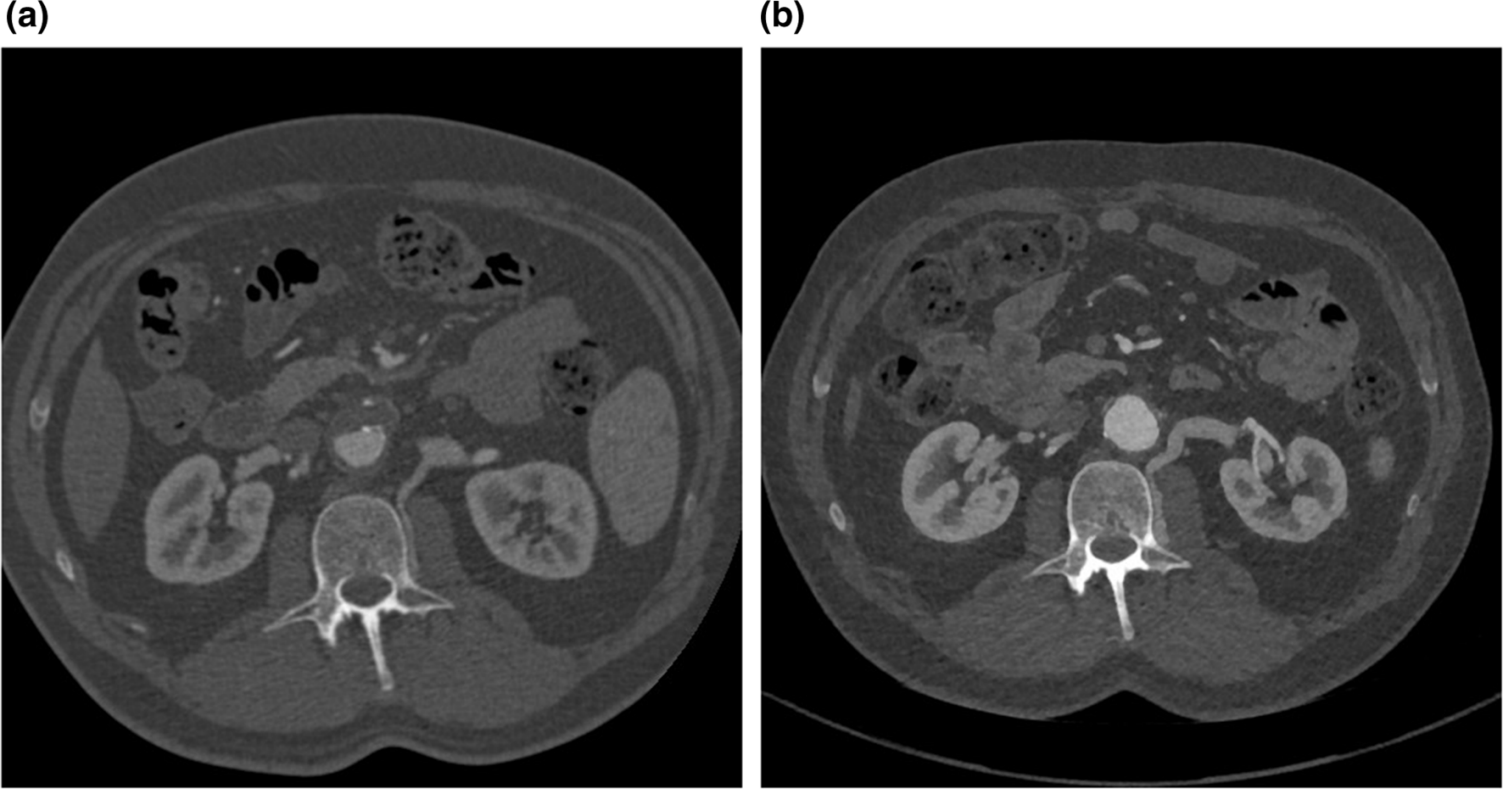
Example of a patient with a strong communicating lumbar vein pre- (**a**) and 5 years post-operatively (**b**)

Our data show that there is no statistically significant difference in short-term perioperative renal function in patients with LRVD compared to patients with LRV mobilization only. The data become interesting when follow-up is evaluated. When groups A and B are compared no differences are observed, however, after following-up for two years, we could identify a cohort of patients with better renal outcome after ligation of the left renal vein. In this group we could confirm a communicating lumbar vein ≥ 2 mm between the left renal vein and the left ascending lumbar vein on pre-operative CT scans. Renal function was significantly better compared to the group without a communicating lumbar vein (*p* = 0.016). There was no difference in patient management between the groups. We suggest that this result is probably linked to better venous drainage from the left kidney offered by a stronger network of venous collaterals that prevents damage to the organ due to congestion (Fig. 1).

Our data suggest that the presence of the communicating lumbar vein may provide an alternative way of drainage that allows restoration of the renal function to preoperative levels as seen in group A+ , we could not observe this recovery in group A−. This has not been described so far and we suppose that recent studies did not find this difference because the communicating lumber vein may not have been considered as a relevant individual factor [7,15].

The accuracy of the venous phase in contrast-enhanced CT to detect venous collaterals or anomalies ranges from 96 to 100% which is excellent and underlines the validity of our observation [8,9,12]. Furthermore, in our cohort of patients, the incidence of the communicating lumbar vein in preoperative contrast-enhanced CT studies was similar to previously reported data (30.9% [8]).

### Limitations

The limitation of our study is the moderate number of patients, due to the low incidence of the disease and a mindful selection of patients. However, this is in large part due to the strict selection criteria in this study as we did not include infrarenal abdominal aortic aneurysms, ruptured abdominal aortic aneurysms or patients who received additional treatment to the renal arteries during juxtarenal aortic repair. We exclusively analysed patients with juxtarenal abdominal aortic aneurysms in whom a suprarenal aortic cross clamp was applied. All our patients were treated via a transperitoneal approach, so we do not have data on the postoperative renal function in patients with juxtarenal aortic aneurysms treated by a retroperitoneal approach, which per se can eliminate the need of LRV division. In case of insufficient exposure after extensive mobilization of the LRV, our centre’s policy is to divide the LRV rather than to divide the adrenal and gonadal and lumbar vein as this procedure enhances the exposure only to a limited extent.

The limited number of patients is also relevant to the observation of a trend towards a better renal function in the early postoperative period in patients in whom the left renal vein could be preserved (*p* = 0.09 and 0.12 on days 10 and 15). With a higher number of patients, this result may reach significance.

Future studies with more patients and longer follow-up periods are needed to evaluate the impact of our findings on the long-term.

## Conclusion

In patients treated for juxtarenal abdominal aortic aneurysms, LRV division appears not to affect renal function in the short-term. The absence of a communicating lumbar vein ≥ 2 mm in the preoperative CT scan in patients requiring LRV division seems to correlate with a deterioration of renal function in the long-term. Therefore, we suggest reserving this procedure if possible to patients presenting a good communicating lumbar vein in the preoperative CT scan, to have an additional route of good venous drainage for a better renal outcome. Otherwise reconstruction of the LRV may be advisable, especially in patients that would be more affected in the long-term from a renal deterioration such as patients with one kidney or already increased creatinine preoperatively. Further data are necessary to corroborate our results.

## References

[1] Mayer D, Pfammatter T, Lachat M (2018) Juxtarenale, suprarenale und Abschnitt IV-Aneurysmen der Aorta: Klinik, Diagnostik und konventionelle Therapie. Debus E, W G-F, (eds) Springer, Heidelberg

[2] Mehta T, Wade RG, Clarke JM (2010). Is it safe to ligate the left renal vein during open abdominal aortic aneurysm repair?. Ann Vasc Surg.

[3] Marrocco-Trischitta MM, Melissano G, Kahlberg A, Setacci F, Segreti S, Spelta S (2007). Glomerular filtration rate after left renal vein division and reconstruction during infrarenal aortic aneurysm repair. J Vasc Surg.

[4] Huber D, Harris JP, Walker PJ, May J, Tyrer P (1991). Does division of the left renal vein during aortic surgery adversely affect renal function?. Ann Vasc Surg.

[5] West CA, Noel AA, Bower TC, Cherry KJ, Gloviczki P, Sullivan TM (2006). Factors affecting outcomes of open surgical repair of pararenal aortic aneurysms: a 10 year experience. J Vasc Surg.

[6] AbuRahma AF, Robinson PA, Boland JP, Lucente FC (1991). The risk of ligation of the left renal vein in resection of the abdominal aortic aneurysm. Surg Gynecol Obstet.

[7] Samson RH, Lepore MR, Showalter DP, Nair DG, Lanoue JB (2009). Long-term safety of left renal vein division and ligation to expedite complex abdominal aortic surgery. J Vasc Surg.

[8] Yao Y, Okada Y, Yamato M, Ohtomo K (2003). Communicating vein between the left renal vein and left ascending lumber vein: incidence and significance on abdominal CT. Radiat Med.

[9] He B, Hamdorf JM (2013). Clinical importance of anatomical variations of renal vasculature during laparoscopic donor nephrectomy. OA Anatomy.

[10] Wukasch DC, Iverson LI, Rubio PA (1976). The left renal lumbar vein: importance in exposure of the renal arteries. Cardiovasc Dis.

[11] Li G, Dong J, Lu JS, Zu Q, Yang SX, Li HZ (2011). Anatomical variation of the posterior lumbar tributaries of the left renal vein in retroperitoneoscopic left living donor nephrectomy. Int J Urol.

[12] Pilcher JM, Padhani AR (1997). Problem in diagnostic imaging: behind the left renal vein. Clin Anat.

[13] Cockcroft DW, Gault MH (1976). Prediction of creatinine clearance from serum creatinine. Nephron.

[14] Xie P, Huang JM, Lin HY, Wu WJ, Pan LP (2013). CDK-EPI equation may be the most proper formula based on creatinine in determining glomerular filtration rate in Chinese patients with chronic kidney disease. Int Urol Nephrol.

[15] Wang L, Xin SJ, Song Z, Zhang J (2013). Left renal vein division during open surgery of abdominal aortic disease: a propensity score-matched case-control study. Eur J Vasc Endovasc Surg.

[16] Pandirajan K, Katsogridakis E, Sidloff D, Sayers RD, Bown MJ, Saratzis A (2020). Effects of left renal vein ligation during open abdominal aortic aneurysm repair on renal function. Eur J Vasc Endovasc Surg.

[17] Stenstrom JD, Ford HS, MacKay MI, Hosie RT, Donald JC (1976). Ruptured abdominal aortic aneurysms. Am Surg.

